# Shellfish Toxin Uptake and Depuration in Multiple Atlantic Canadian Molluscan Species: Application to Selection of Sentinel Species in Monitoring Programs

**DOI:** 10.3390/toxins13020168

**Published:** 2021-02-22

**Authors:** Wade A. Rourke, Andrew Justason, Jennifer L. Martin, Cory J. Murphy

**Affiliations:** 1 Dartmouth Laboratory, Canadian Food Inspection Agency, 1992 Agency Drive, Dartmouth, NS B3B 1Y9, Canada; Cory.Murphy@canada.ca; 2 New Brunswick Operations, Canadian Food Inspection Agency, 99 Mount Pleasant Road, P.O. Box 1036, St. George, NB E5C 3S9, Canada; Andrew.Justason@canada.ca; 3 St. Andrews Biological Station, Fisheries and Oceans Canada, 125 Marine Science Drive, St. Andrews, NB E5B 0E4, Canada; Jennifer.Martin@dfo-mpo.gc.ca

**Keywords:** shellfish, marine toxins, monitoring, phytoplankton, sentinel species

## Abstract

Shellfish toxin monitoring programs often use mussels as the sentinel species to represent risk in other bivalve shellfish species. Studies have examined accumulation and depuration rates in various species, but little information is available to compare multiple species from the same harvest area. A 2-year research project was performed to validate the use of mussels as the sentinel species to represent other relevant eastern Canadian shellfish species (clams, scallops, and oysters). Samples were collected simultaneously from Deadmans Harbour, NB, and were tested for paralytic shellfish toxins (PSTs) and amnesic shellfish toxin (AST). Phytoplankton was also monitored at this site. Scallops accumulated PSTs and AST sooner, at higher concentrations, and retained toxins longer than mussels. Data from monitoring program samples in Mahone Bay, NS, are presented as a real-world validation of findings. Simultaneous sampling of mussels and scallops showed significant differences between shellfish toxin results in these species. These data suggest more consideration should be given to situations where multiple species are present, especially scallops.

## 1. Introduction

Shellfish toxins have been present and monitored for many decades on the Canadian Atlantic coast [1]. The first North American shellfish sanitation regulations came into effect in 1925 [2], and a Canada/USA bilateral agreement on shellfish sanitation was enacted in 1948 [3]. This agreement is still in place, and the key principles are delivered through the National Shellfish Sanitation Program (NSSP) in the USA and the Canadian Shellfish Sanitation Program (CSSP) in Canada. The CSSP is delivered by three government departments: the Canadian Food Inspection Agency (CFIA), Environment and Climate Change Canada (ECCC) and Fisheries and Oceans Canada (DFO). The CFIA performs marine toxin monitoring for paralytic shellfish toxins (PSTs), amnesic shellfish toxin (AST), and lipophilic shellfish toxins (LSTs). Health Canada has established maximum limits (MLs) for these toxins in bivalve shellfish edible tissue [4]: 0.8 mg saxitoxin (STX) equivalents/kg for PSTs, 20 mg/kg domoic acid (DA) for AST, 0.2 mg okadaic acid (OA) equivalents/kg and 0.2 mg pectenotoxin (PTX)/kg for LSTs. These toxins have all been responsible for Canadian harvest area closures from time to time when shellfish concentrations have exceeded an ML [5]. A thorough review of occurrence, modes of action and chemical properties for these toxins is included in Daneshian et al. [6].

Laboratory methods available to detect and quantify these toxins have changed considerably over time. Monitoring of PSTs was originally completed using a mouse bioassay method [7], but now there are multiple chemistry-based analytical methods [8,9] and a receptor-binding assay [10] that have been validated and approved as AOAC Official Methods of Analyses. Additionally, LC-MS/MS methods have now been validated for PST analysis [11,12] and offer even greater selectivity and confirmation ability. Monitoring of AST has been performed consistently with chemistry-based analytical methods [13], with improvements as technology has advanced [14]. Monitoring of LSTs, like PSTs, was previously widely performed using a mouse bioassay [15], but has now advanced to analytical methods using LC-MS/MS [16]. The use of chemistry-based analytical methods requires purified standards for each individual toxin, and known toxic equivalence factors (TEF) in order to calculate results. Despite these additional needs, the lower detection limits and toxin profile information that these methods provide are invaluable in modern monitoring programs [17,18].

**Figure 1:**
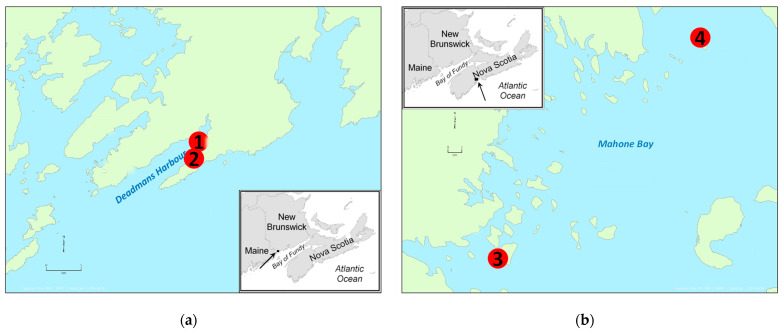
Maps of sampling areas: (**a**) Deadmans Harbour, NB; (**b**) Mahone Bay, NS. Sampling sites are labelled as follows: 1. Deadmans Harbour natural clam bed, 2. Deadmans Harbour experimental site for submerged cages, 3. Indian Point, 4. Snake Island.

Phytoplankton monitoring results have the potential to be used as an early warning for elevated toxin levels in shellfish, although there are many variables that are not well understood with regard to phytoplankton population dynamics and toxin production [19,20]. Many different species of phytoplankton can cause toxin outbreaks, for example, *Alexandrium catenella* has been responsible for producing PSTs in Deadmans Harbour, NB, (Figure 1a) in the Bay of Fundy [19,21] and *Pseudo-nitzschia pseudodelicatissima* has been responsible for producing AST in the Bay of Fundy [22]. Some countries monitor phytoplankton counts as part of routine shellfish monitoring programs [23,24], although it is not required as part of the CSSP.

There have been studies examining toxin accumulation and depuration of PSTs, AST, and LSTs in shellfish; some of these studies have involved opportunistic sampling during toxin blooms [25,26,27,28,29], while others have been controlled laboratory studies where shellfish were fed toxic phytoplankton [28,30,31,32,33,34,35,36,37]. These studies have described observations in single species, and in some cases, included comparisons between species. The diversity and complexity of shellfish environments mean that these studies cannot fully describe the processes of toxin accumulation and depuration in the natural environment. Other studies have suggested ways to mitigate the impact of toxic episodes, such as methods for decreasing toxin accumulation [38,39] and increasing depuration rates [40]. These techniques may not be practical on a large scale, are generally only applicable to aquaculture settings, and have not negated the need for routine monitoring programs.

The CSSP has been very effective, with only a single documented outbreak of shellfish-toxin related illnesses associated with legally harvested shellfish in recent history [41]; however, efforts are always being made to improve monitoring. It is important to ensure that the risk of elevated toxin levels is adequately assessed in each harvesting area, and mussels (*Mytilus spp.*) are the most common species used for this purpose in Canada and other areas [23]. Monitoring toxin levels in multiple shellfish species simultaneously poses a difficulty in assessing risk in shellfish harvest areas. Furthermore, the unpredictable nature of toxin-producing algal blooms makes it difficult to plan experiments to gather information to develop strategies to address these issues.

This paper describes results from a combination of (1) a designed research project and (2) opportunistic sampling. The objective of the designed research project was to validate the use of mussels as the sentinel species for monitoring PSTs (and represent the highest PST risk in various bivalve species). The study was completed in Deadmans Harbour, NB, where historic monitoring results demonstrated the annual presence of both *A. catenella* cells and PSTs in shellfish; PSTs are the most prevalent shellfish toxins detected in eastern Canada, and there are few sites with this level of predictability. Although *P. pseudodelicatissima* was observed annually at the test site, the shellfish rarely accumulated AST. This experiment was later expanded when AST was detected in routine monitoring of shellfish samples. Blue mussels (*Mytilus edulis*), soft-shell clams (*Mya arenaria*), Atlantic sea scallops (*Placopecten magellanicus*), and eastern oysters (*Crassostrea virginica*) from other harvest areas were stored in, and subsequently simultaneously harvested from, submerged cages 150–250 m distance from the natural clam bed (a routine CSSP monitoring station). All shellfish were harvested from other areas, tested to ensure that they contained no toxins when transferred, and then allowed to acclimate to conditions in Deadmans Harbour for at least 3 weeks before being sampled. PST results were used to compare accumulation and depuration rates between species. Water samples were also collected in close proximity to the suspended cages and the total phytoplankton community was analyzed. Species enumeration included cell counts for *A. catenella,* which were compared with PST levels in shellfish to assess the application of phytoplankton monitoring as a predictor of PSTs in shellfish.

Additional data were obtained from opportunistic sampling at two harvest sites in Mahone Bay, NS, (Figure 1b) when mussels and scallops were sampled simultaneously due to elevated toxin levels noted at these sites. This sampling was a combination of planned monitoring samples and targeted sampling in response to increasing toxin levels. As the generation of these data was not specifically designed to support this paper, not all species were sampled at each time point. These data are presented as validation of the results from the research study. Table 1 shows the number of samples collected at each site and which toxin groups were analyzed.

Despite the length of time that routine monitoring has been in place around the world, much remains to be understood about the toxin accumulation and depuration rates of various species [28,42,43,44,45]. This paper contributes information about the toxin uptake of multiple species, which can be used to improve the design and implementation of shellfish toxin monitoring programs.

## 2. Results

### 2.1. Research Project—Deadmans Harbour, NB

#### 2.1.1. Toxin Monitoring in Shellfish

**Figure A1:**
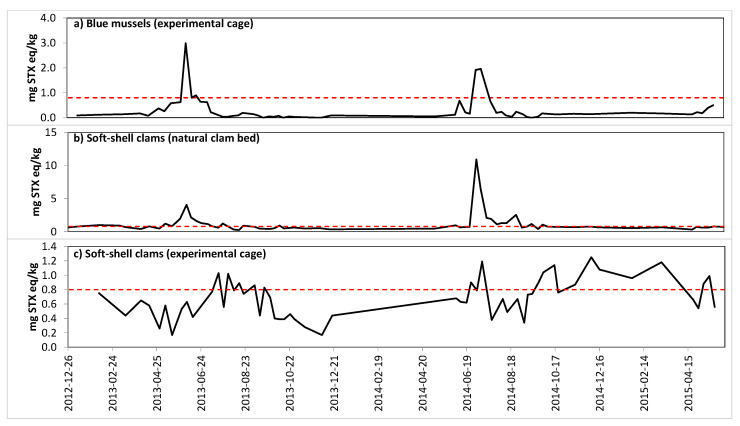
Total PST concentrations of mussels and clams from Deadmans Harbour (**a**) blue mussels (experimental cage), (**b**) soft-shell clams (natural clam bed) and (**c**) soft-shell clams (experimental cage). The dashed red lines represent the Canadian PST ML.

Results from PST analyses of clams held in submerged cages were inconsistent with the results from all other species in this study and did not demonstrate any peak in toxin level (Figure A1). This could be explained by a number of factors, including that the clams were not in their natural environment (tidal mud flats), experienced more turbulent oceanographic conditions, or had altered feeding rates due to stocking density or fouling of cages. Appendix A includes a detailed rational for excluding soft-shell clam results from this study, including analysis of long-term comparison of mussel and soft-shell clams at another CSSP monitoring site (Table A1). The original study design was to compare PST concentrations between samples from experimental cages, and to use the sample results from the natural clam bed as validation of the results; instead, the results from the natural clam bed were used to exclude soft-shell clam data.

**Figure 2:**
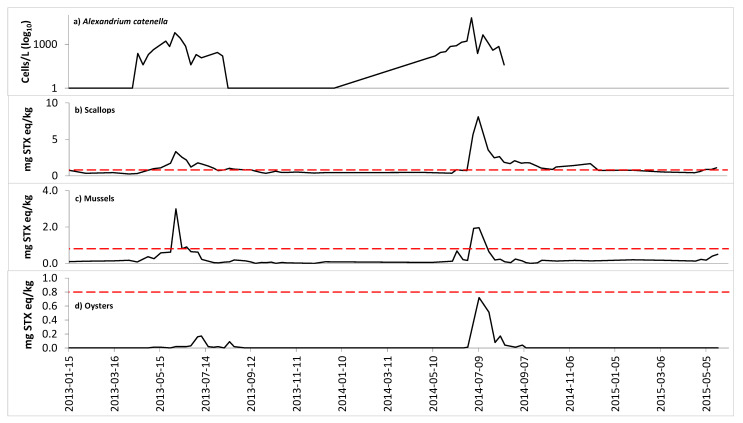
Cell counts (cells/L) for (**a**) *Alexandrium catenella* and total paralytic shellfish toxins (PST) concentrations (mg STX eq/kg) for species harvested from experimental cages in Deadmans Harbour, NB (**b**) scallops; (**c**) mussels; (**d**) oysters. The dashed red lines represent the Canadian PST maximum limit (ML).

Figure 2 shows PST results from multiple shellfish species. All species accumulated some PSTs during the project, but oysters were the only species in which PST concentrations never exceeded the ML. The PST concentrations in mussels changed rapidly and coincided with the rise and fall of adjacent *A. catenella* cell counts (Figure 2). Scallop samples exceeded the ML before mussel samples during the toxic episodes in 2013 and 2014, and provided the earliest warning of increasing PST levels. Analysis of shellfish before the project began confirmed no PSTs were present in any species, and eliminated the possibility of contamination from previous toxic episodes.

**Figure 3:**
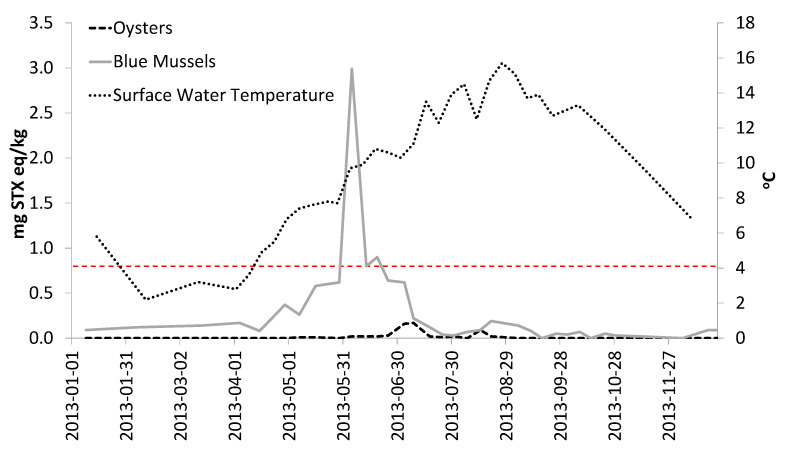
Total PST concentrations (mg STX eq/kg) in mussels and oysters harvested from experimental cages in Deadmans Harbour, NB, with accompanying surface water temperature (°C) for 2013. The dashed red line represents the Canadian PST ML.

Oyster PST concentrations remained significantly lower than mussels and scallops, and there was a delay in observable PST concentrations in oysters relative to mussels and scallops. Figure 3 shows that the PST concentrations in mussels began to increase as surface water temperatures increased in late April/early May.

**Figure 4:**
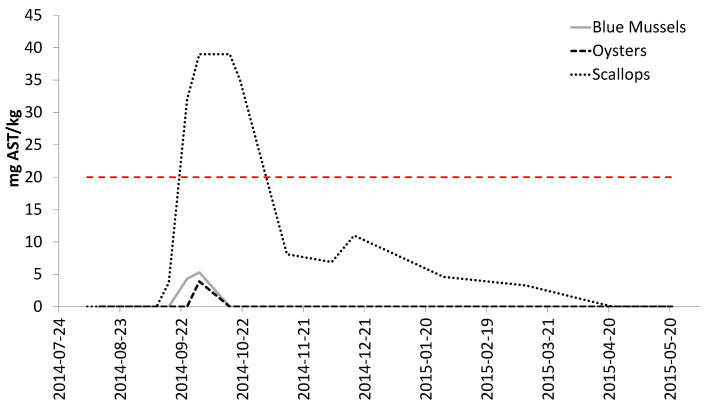
AST concentrations (mg AST/kg) for shellfish harvested from experimental cages in Deadmans Harbour, NB. The dashed red line represents the Canadian AST ML.

The highest AST levels were detected in scallops, which were the only species to exceed the ML (Figure 4). Mussel samples also contained AST, but for a much shorter duration: 6 days in mussels vs. 175 days in scallops. Only a single oyster sample had detectable AST levels during the same time period.

#### 2.1.2. Phytoplankton Monitoring

The total phytoplankton community was analyzed as part of a long-term dataset initiated in 1988, which provided the opportunity to capture a weekly picture of species initiation, development, and decline [46]. The subset of *A. catenella* cell concentrations showed a strong temporal correlation with PST presence in shellfish (Figure 2), while cell counts showed no correlation with PST concentrations in shellfish, as has been observed in previous Bay of Fundy studies [19,47]. Low levels of PSP toxicity can be detected at very low concentrations of A. catenella (20–40 cells/L). The *A. catenella* cell counts changed more rapidly than the shellfish toxin levels. This may have been due to physical oceanography, bloom (duration, intensity and toxicity), very low numbers resulting in shellfish toxicity, patchiness of the cell distributions, the fact that *A. catenella* is often not the dominant phytoplankton species in the community and shellfish can selectively feed on other species, and/or retention and conversion of toxins in shellfish for extended periods. Weekly sampling for *A. catenella* indicates that this frequency of sampling is sufficient to provide an indication of increasing PSTs in tissues. Following the bloom, an absence of *A. catenella* cells within the water column indicates that the shellfish have the potential to depurate and PST concentrations can decrease. This absence of cells can act as a signal to increase PST analyses in order to measure the decline in toxins and determine the timing for the safe marketing of shellfish. *Pseudo-nitzschia pseudodelicatissima* cell counts were not available for 2014.

### 2.2. Validation of Research Findings with Routine Monitoring Samples—Mahone Bay, NS

**Figure 5:**
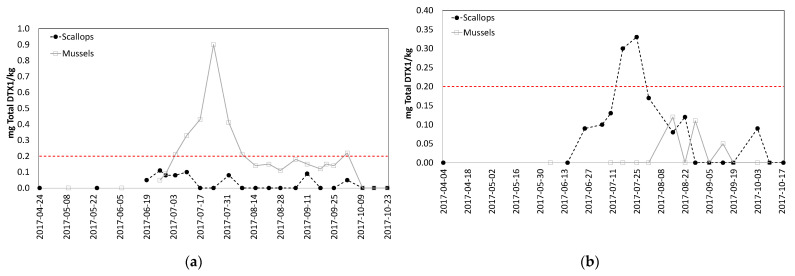
LST concentrations (mg total DTX1/kg) for shellfish harvested in Mahone Bay, NS: (**a**) Indian Point, NS; (**b**) Snake Island, NS. The dashed red lines represent the Canadian LST ML.

Samples obtained from two sites in Mahone Bay, NS, highlighted significant differences in LST concentrations between scallops and mussels. At one site (Indian Point), LST levels were 10 × higher in mussels than in scallops, while at the other site (Snake Island), scallop LST levels were higher (Figure 5). It is also noteworthy that scallops did not retain LST for an extended time as they did for PST and AST.

**Figure 6:**
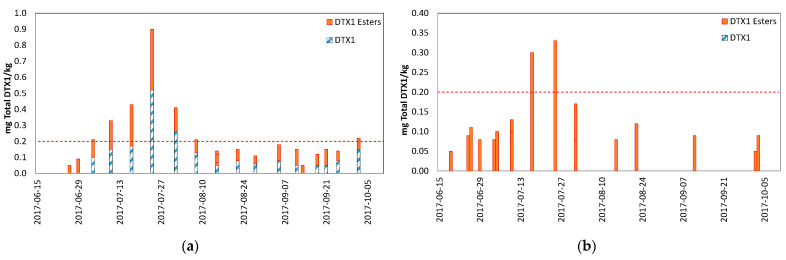
LST toxin profile concentrations (mg total DTX1/kg) for shellfish harvested in Mahone Bay, NS (Indian Point and Snake Island combined): (**a**) mussels; (**b**) scallops. The dashed red lines represent the Canadian LST ML.

The only LST toxins detected were dinophysistoxin-1 (DTX1) and DTX1 esters; no OA or DTX2 were detected. Mussel samples were contaminated with both free and esterified forms of DTX1, with esterified forms contributing an average of 51% of the total toxicity (ranging from 32–100%) (Figure 6). No free DTX1 was detected in scallops; all toxins observed in scallops were present in the esterified form. The data in Figure 6 are displayed by species (combination of Indian Point and Snake Island samples); no differences in esterification rates were detected between those sites.

**Figure 7:**
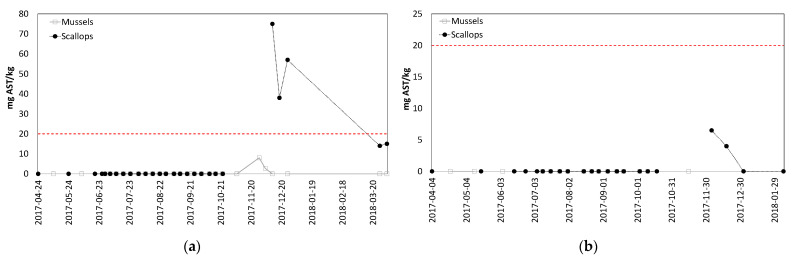
AST concentrations (mg AST/kg) for shellfish harvested in Mahone Bay, NS: (**a**) Indian Point, NS; (**b**) Snake Island, NS. The dashed red lines represent the Canadian AST ML.

The AST results from Mahone Bay, NS are presented in Figure 7. The onset of toxicity in scallops was not captured, because mussels and scallops were not sampled simultaneously until AST was detected. These data show that scallops accumulated higher AST concentrations than mussels, and scallops also retained the toxin over a much greater period of time than mussels. Scallops and mussels were both tested and found to have no toxins one month before AST was detected (markers visible on x-axis indicate sampling events when no toxins were detected); this is significant because it confirms that AST detected in shellfish were a result of a new contamination, not residual levels from previous exposures.

Raw data from all figures is available in the Supplementary Materials.

## 3. Discussion

The PST concentrations in mussels changed rapidly in comparison to the other species in this study and coincided with the rise and fall of *A. catenella* cell counts. This rapid rise and subsequent fall of PST levels in mussels suggests that they tend to accumulate and depurate the toxins more rapidly than other species, which greatly increases the possibility of missing spikes in toxin levels, because the window of time with elevated mussel PST levels can be quite narrow; this is consistent with previous observations [17]. A spike in toxin levels could occur between PST sampling events and be missed if mussels were the only species sampled. However, weekly *A. catenella* sampling would indicate the presence and magnitude of *A. catenella* cell concentrations, thus providing warning of PST shellfish toxicity so that shellfish sampling and analysis could be increased. The peak PST concentration in mussels from this study was higher in 2013 than 2014, but the opposite trend was observed in scallops and oysters from the submerged cages and clams from the natural clam bed (Appendix A). Since mussel PST levels change rapidly, this may indicate that a mussel sample was collected in 2014 just before or after a rapid change in PST concentrations, but before the concentrations changed in the other species. The observation of extended PST retention in scallops compared to mussels suggests that scallops may need to be monitored, in addition to mussels, to reopen harvest areas when scallops are present and being harvested.

Scallops were the first species with detectable levels of AST, with 1-week and 2-week delays before AST levels were observed in mussels and oysters, respectively. Scallops also retained AST longer than mussels; this is consistent with published literature documenting retention of AST in scallops [20,25,30,31], as well as rapid depuration of AST from mussels [20,26,30]. These AST results further support the conclusion that scallops may need to be monitored, in addition to mussels, when scallops are being harvested.

A delay was observed in the rise of PST levels in oysters relative to mussels and scallops. This delay could be related to the temperature dependence of oyster feeding behavior [33], as this study confirmed that oysters did not accumulate PSTs when the surface water temperature was <10 °C, consistent with previous research [33]. Recent research [34,35,48,49] with other shellfish species suggests that PST accumulation and depuration rates could be significantly different (lower toxin accumulation rates and slower depuration rates) at warmer temperatures. The effect of increased surface water temperatures on shellfish accumulation and depuration rates will need to be explored with relevant shellfish species to determine if there could be a potential future impact on shellfish monitoring in eastern Canada.

The use of phytoplankton monitoring as an early PST warning was also considered. *Alexandrium catenella* cell counts are predictive of shellfish PST levels in some areas [50,51], but in other areas, this relationship is only qualitative and cannot be used to predict PST levels in shellfish tissue [19,52]. The current study confirmed that *A. catenella* cell counts are not predictive of shellfish PST levels in the Bay of Fundy, since there was no delay between the peak *A. catenella* cell counts and peak shellfish toxicity. This was not unexpected, as it is rare for peak *A. catenella* cell abundance to show a correlation with shellfish toxicity in the Bay of Fundy [19]. However, *A. catenella* monitoring can provide valuable complementary data for PST monitoring programs and industry in Atlantic Canada, but based on this limited data set and previous work in the Bay of Fundy [19], it is not in a position to replace or allow for reduced shellfish sampling at this particular monitoring site (and is not included in CSSP monitoring). Other work has reached a similar conclusion for LSTs [53]. In addition, higher cell counts increase the potential for toxic episodes, but they are not always predictive since some phytoplankton species do not produce toxins consistently, and higher toxin production can sometimes be observed with relatively low cell counts [19]. It is noteworthy that *A. catenella* from the Bay of Fundy always produce toxins; however, other factors impact shellfish uptake rates (such as position in the water column, weather conditions, physical oceanography of an area, etc.). These data do not diminish the value of phytoplankton monitoring to add context to PST results (especially when PST levels in shellfish are below the ML); elevated cell counts still indicate an increased potential for toxin production, which could be used as a trigger for targeted sampling (or other actions).

An opportunity to validate conclusions from the research project was presented when AST and LST concentrations rose in Mahone Bay, NS during 2017. Toxin levels were elevated at harvest sites with both mussels and scallops during the summer and fall, and resulted in sampling of both species. The simultaneous sampling of both species necessary to confirm the relative accumulation and depuration rates was not conducted because this was reactive sampling completed to ensure food safety, not a designed research project; only one species was sampled during some weeks. The conclusions based on AST results from the research project at Deadmans Harbour were confirmed by these real-life monitoring results; scallops accumulated higher AST levels and retained the toxin longer than mussels. Results from LST analyses also demonstrated that a single species cannot always represent the risk for other species, as mussels were the higher risk species at one site (Indian Point), while scallops were the higher risk species at the other site (Snake Island). Samples of both species were tested and found to contain no toxins within a month of onset of this toxic episode. These results make it particularly difficult to select a single species for routine monitoring. The fact that LST levels in scallops at Indian Point never exceeded the ML is noteworthy, as is the fact that the LST levels were present in mussels for longer than scallops. This is consistent with LST observations in different mussel and scallop species [27], although different than the toxin retention behavior observed in scallops for PSTs [1] and AST [25,30,31]. A difference was also observed in the LST toxin profiles for mussels and scallops. LSTs detected in mussels were both free and esterified forms of DTX1; the esterified forms of DTX1 contributed an average of 51% to the total toxicity (ranging from 32–100%). No free DTX1 was detected in scallops from either site; all toxins observed in scallops were present only in the esterified form. This highlights the differences in toxin profiles between species [45], as well as the importance of performing the alkaline hydrolysis step necessary to liberate the esterified forms of these toxins during analysis. There could be a significant under-estimation of risk if scallops were selected as the sentinel species and analyzed without looking for the esterified forms of LSTs.

These data all demonstrate that a single species cannot always represent the risk accurately when multiple shellfish species are present in a harvest area; Bresnan et al. reached the same conclusion [20]. The routine monitoring data from Mahone Bay presented cannot fully validate the conclusions of the research project because there were no PST levels detected in shellfish sampled for routine monitoring at these times; however, when all the available data are considered together, they highlight that different approaches may be needed to deal with different species and risks associated with different toxins. The risks in all situations included in this paper, and especially those in Mahone Bay, were managed through routine CSSP procedures; harvest areas were closed appropriately and no illnesses were linked with any of these results. Mussels are a hardy species, easily maintained in cages, present at many harvest areas, and easily sampled; all these factors support using mussels for toxin monitoring. The presented data do not suggest that mussels should not be used for monitoring, but that the appropriate context should be considered when interpreting toxin levels in mussels. This is especially true in areas with multiple shellfish species present. As an example, a sampling procedure employed in Mahone Bay, NS, was to sample both species regularly, alternating between mussels and scallops until toxins were detected, and then to sample both species simultaneously to ensure that the highest risk was identified. This is also consistent with the decision by CFIA in BC to sample geoducks and mussels when both species are present, because data have demonstrated that PST levels in mussels do not represent PST levels in geoducks [54].

## 4. Conclusions

Mussels have been used as the sentinel species in Atlantic Canadian shellfish toxin monitoring programs for many years. These samples and programs have generally protected consumers from illnesses related to shellfish toxins. Additional context may be necessary if there are multiple species in the same harvest area, where additional species may need to be sampled along with mussels to evaluate the food safety risk associated with each species.

## 5. Materials and Methods

All testing was completed using validated methods in an ISO 17025 accredited laboratory.

### 5.1. Samples

Shellfish (blue mussels, soft-shell clams, Atlantic sea scallops, and eastern oysters) were harvested from other areas, tested to ensure they contained no toxins when transferred, and then allowed to acclimate to conditions in Deadmans Harbour, NB for at least 3 weeks before being sampled. Shellfish were kept in submerged cages on the ocean floor for the research project in Deadmans Harbour, NB (2013–2015). Shellfish were sampled simultaneously, and sampling frequency ranged from weekly in summer to monthly in winter, according to PST risk and CSSP sampling frequency.

The retention of PSTs in various scallop tissues has been documented for a long time [1,55,56]. Atlantic sea scallop (*Placopecten magellanicus*) adductor muscles are commonly consumed in Canada (not whole scallops) and lower (or no) PST levels are present in Atlantic sea scallop adductor muscle compared to whole scallops which contain digestive materials that accumulate toxins. Whole scallops were analyzed in this study to assess toxin concentrations, and because whole tissue is the easiest tissue to use for regulatory monitoring.

Samples of mussels and scallops were obtained from aquaculture operations in Mahone Bay, NS in support of CSSP monitoring.

Water samples (250 mL) were collected weekly (although not always on the same day as shellfish samples) for phytoplankton analysis from the surface in close proximity to the suspended cages and preserved with 2.5% formalin acetic acid (FAA) (Fisher Scientific, Nepean, ON, Canada). Later, 50 mL subsamples were settled in counting chambers for 16 h and the whole surface area was counted and enumerated for total phytoplankton community, including *A. catenella* and *P. pseudodelicatissima* concentrations (as cells/L or chains of cells/L) using a Nikon inverted microscope (Nikon Instruments, Melville, NY, USA). A vertical 20 µm mesh phytoplankton 30 cm net sample was collected, preserved with FAA for qualitative analysis of dominant phytoplankton (as well as harmful) species using a compound Nikon microscope (Nikon Instruments, Melville, NY, USA).

### 5.2. Reagents and Chemicals

Instrument solvents, test reagents, and chemicals for the analysis of all sample types were either HPLC- or LC-MS-grade, as appropriate for the assay. Certified reference materials (CRMs) used for preparing instrumental calibrants were all obtained from Biotoxin Metrology, NRCC, Halifax, Canada.

### 5.3. Sample Preparation

Samples of shellfish were shucked and extracted following internal laboratory protocols prior to analysis for marine toxins. Subsamples of the tissue homogenates were extracted as noted below. Whole shellfish tissue was analyzed in all cases.

### 5.4. PST

All PST testing was conducted on 5 ± 0.25 g subsamples of tissue homogenate by LC with post-column oxidation and fluorescence detection (LC-PCOX-FLD) following AOAC OMA 2011.02 [9] using single-point calibration [57,58]. Analyses were carried out with Agilent 1200 LC systems (Agilent Technologies, Kirkland, PQ, Canada) and Waters reagent manager pumps and post-column reaction modules (Waters Limited, Milford, MA, USA) fitted with 1.0 mL knitted teflon reaction coils (Sigma-Aldrich Canada, Oakville, ON, Canada). PST analogues included in the method were saxitoxin (STX), neosaxitoxin (NEO), decarbamoyl saxitoxin (dcSTX), gonyautoxins 1 to 5 (GTX1–5), decarbamoyl gonyautoxins 2 and 3 (dcGTX2&3), and N-sulfocarbamoyl gonyautoxins 2 and 3 (C1&2). PST method LOD estimates are shown in Table 2.

### 5.5. AST

All AST testing was conducted on subsamples of tissue homogenate by LC-UV using an in-house method based on [13,14]. Analyses were carried out with an Agilent 1290 UHPLC system (Agilent Technologies, Kirkland, PQ, Canada) with UV-diode array detection. Tissue homogenate was weighed (5 ± 0.25 g) into a 50 mL polypropylene centrifuge tube. Then, 5.0 mL water was added, the mixture was vortexed before adding 10.0 mL methanol (Caledon Laboratories, Georgetown, ON, Canada). The mixture was vortexed again and then centrifuged at ≥1000 g for 10 min. Approximately 1.5 mL resulting supernatant was filtered through a 0.2 µm nylon syringe filter. Filtered sample extract (750 µL) was transferred to an autosampler vial and diluted with 750 µL water, and vortexed. Injections of 2 µL were performed on a Waters Acquity UPLC BEH C18, 1.7 µM, 2.1 × 50 mm column (Waters Limited, Taunton, MA, USA) at 50 °C, and a flow rate of 0.7 mL/min. Mobile phase A was water + 0.1% formic acid (Sigma-Aldrich Canada, Oakville, ON, Canada), and mobile phase B was acetonitrile (Caledon Laboratories, Georgetown, ON, Canada) + 0.1% formic acid. AST was eluted during a 1.2 min isocratic hold at 8% mobile phase B, and this was followed by a 0.5 min isocratic hold at 50% mobile phase B to flush the column, and 0.3 min isocratic hold at 8% mobile phase B to re-equilibrate at starting conditions for the next injection. AST peaks were measured at 242 nm and confirmed by spectral comparison with external calibration standards. The method LOD was 0.7 mg/kg shellfish tissue. AST analogues included domoic acid and epi-domoic acid.

### 5.6. LST

All LST testing was conducted on 2 ± 0.05 g subsamples of tissue homogenate by LC-MS/MS [59] with no SPE cleanup and separation on a Waters Acquity UPLC BEH Shield RP18, 1.7 μm, 2.1 × 100 mm column (Waters Limited, Taunton, MA, USA) with acidic mobile phase [16]. Analyses were carried out with either an Agilent 1290 UHPLC system (Agilent Technologies, Kirkland, PQ, Canada) coupled to an AB Sciex 5500 QTrap MS/MS (AB Sciex, Concorde, ON, Canada) or a Waters I-class UPLC coupled to a Waters Xevo TQ-S Micro MS/MS (Waters Limited, Millford, MA, USA). LST analogues included in the method were gymnodimine (GYM), pinnatoxins A, E, F, and G (PnTX-A, PnTX-E, PnTX-F, PnTX-G), PnTX esters, azaspiracids 1 to 3 (AZA1-3), okadaic acid (OA), OA esters, dinophysistoxins 1 to 2 (DTX1-2), DTX1-2 esters, yessotoxin (YTX), homoYTX, 45-OH YTX, and 45-OH homoYTX. LST method LOD estimates are shown in Table 3.

## Figures and Tables

**Table 1:** Sampling locations and numbers of samples included in this study for paralytic shellfish toxins (PST), amnesic shellfish toxin (AST), and lipophilic shellfish toxins (LST).

Toxin	Harvest Site	Mussels	Scallops	Oysters
PST	Deadmans Harbour, NB ^1^	63	63	62
AST	Deadmans Harbour, NB ^1^	20	20	19
AST	Indian Point, NS ^2^	26	25	− ^4^
AST	Snake Island, NS ^2^	16	21	− ^4^
LST	Indian Point, NS ^3^	22	22	− ^4^
LST	Snake Island, NS ^3^	13	17	− ^4^

^1^ Sampled January 2013–May 2015; ^2^ Sampled April–December 2017; ^3^ Sampled April–October 2017; ^4^ No oysters at these sites.

**Table 2:** LOD estimates for compounds in PST method (µg STX eq/kg).

PST Analogue	GTX4	GTX1	dcGTX3	GTX5	dcGTX2	GTX3	GTX2	NEO	dcSTX	STX	C1	C2
LOD (µg STX eq/kg)	10	25	1.1	5.1	3.5	1.5	7.5	19	11	11	0.3	1.4

**Table 3:** LOD estimates for regulated compounds in LST method (ng/g).

LST Analogues	AZA1	AZA2	AZA3	DTX1	DTX2	OA	PTX2	YTX
LOD (ng/g)	0.5	0.5	0.4	49	17	23	0.8	50

## Data Availability

The data presented in this study are available in supplementary material.

## Supplementary Materials

The following are available online at https://www.mdpi.com/2072-6651/13/2/168/s1, Excel: Sentinel species supplemental information.

## Appendix A

Figure A1 shows total PST concentrations of clams from the natural bed and mussels and clams from the experimental cages 150–250 m away. The fact that mussel results consistently remained lower than soft-shell clam results (a vs b) throughout the 2-year project was unexpected. Mussels are sampled at a lower frequency than soft-shell clams, because it is illegal to harvest mussels in that area, and soft-shell clams are the primary species used to monitor PST concentrations. Mussel sampling is maintained at some sites to monitor differences between species, and as a potential early warning of toxic episodes. A 15-year dataset from routine monitoring at a nearby harvest site, Lepreau Basin, NB was analyzed for trends between PST levels in soft-shell clams and mussels. These data are summarized in Table A1. The fact that mussels had the higher PST concentration in 67% of simultaneous sampling events, and that the difference between PST concentrations in mussels and soft-shell clams was much larger when mussels had higher PST concentration both suggest that soft-shell clam PST concentrations are generally lower than mussel PST concentrations, and very rarely are soft-shell clam concentrations significantly higher than mussel PST concentrations. This called the validity of the current data into question.

Figure A1 (b vs. c) shows total PST concentrations of clams from the natural bed and clams from the experimental cages 150–250 m away. There is a large difference between results, with clams from the experimental cages never demonstrating a peak in total PST concentration; the concentration remained consistent throughout the project. These data support the conclusion that soft-shell clam samples in this study were not representative, and should be removed from further data analysis.

**Table A1:** Summary of mussel and clam sampling from Lepreau Basin, NB from 2000–2015.

Description	Mussels	Clams
Total samples	129	615
Sampling events when both species were collected simultaneously	89	
Correlation of PST concentration between mussels and clams	0.84	
Samples with highest PST concentration	60 ^1^	15 ^1^
PST range (mg STX equiv/kg)	0.04–37.2	0.12–9.29
Largest PST difference (mg STX equiv/kg)	33.9 ^2^	0.75 ^3^
Average PST difference (mg STX equiv/kg)	2.76 ^2^	0.25 ^3^

^1^ 14 sampling events had equal PST concentrations for both species; ^2^ when mussels had highest PST concentration; ^3^ when clams had highest PST concentration.
